# Elucidating early CT after pancreatico-duodenectomy: a primer for radiologists

**DOI:** 10.1007/s13244-018-0616-3

**Published:** 2018-04-13

**Authors:** Massimo Tonolini, Anna Maria Ierardi, Gianpaolo Carrafiello

**Affiliations:** 1 Department of Radiology, “Luigi Sacco” University Hospital, Via G.B. Grassi 74, 20157 Milan, Italy; 2 Diagnostic and Interventional Radiology Department, ASST Santi Paolo e Carlo, Via A di Rudinì 8, 20142 Milan, Italy

**Keywords:** Pancreatic carcinoma, Pancreatico-duodenectomy, Complications, Pancreatic fistula, Computed tomography (CT)

## Abstract

**Abstract:**

Pancreatico-duodenectomy (PD) represents the standard surgical treatment for resectable malignancies of the pancreatic head, distal common bile duct, periampullary region and duodenum, and is also performed to manage selected benign tumours and refractory chronic pancreatitis. Despite improved surgical techniques and acceptable mortality, PD remains a technically demanding, high-risk operation burdened with high morbidity (complication rates 40–50% of patients). Multidetector computed tomography (CT) represents the mainstay modality to rapidly investigate the postoperative abdomen, and to provide a consistent basis for an appropriate choice between conservative, interventional or surgical treatment. However, radiologists require familiarity with the surgically altered anatomy, awareness of expected imaging appearances and possible complications to correctly interpret early post-PD CT studies. This paper provides an overview of surgical indications and techniques, discusses risk factors and clinical manifestations of the usual postsurgical complications, and suggests appropriate techniques and indications for early postoperative CT imaging. Afterwards, the usual, normal early post-PD CT findings are presented, including transient fluid, pneumobilia, delayed gastric emptying, identification of pancreatic gland remnant and of surgical anastomoses. Finally, several imaging examples review the most common and some unusual complications such as pancreatic fistula, bile leaks, abscesses, intraluminal and extraluminal haemorrhage, and acute pancreatitis.

**Teaching Points:**

*• Pancreatico-duodenectomy (PD) is a technically demanding surgery burdened with high morbidity (40–50%).*

*• Multidetector CT is the mainstay technique to investigate suspected complications following PD.*

*• Interpreting post-PD CT requires knowledge of surgically altered anatomy and expected findings.*

*• CT showing collection at surgical site supports clinico-biological diagnosis of pancreatic fistula.*

*• Other complications include biliary leaks, haemorrhage, abscesses and venous thrombosis.*

## Introduction

Pancreatico-duodenectomy (PD) represents the standard surgical treatment for tumours of the pancreatic head, distal common bile duct, periampullary region and duodenum, and is the only curative option for malignancies. Despite improved surgical techniques and perioperative care, PD remains a technically demanding, high-risk operation that includes complex resections and multiple anastomoses. In the last decade, at high-volume centres the postsurgical mortality after PD dropped below 2–3%. However, PD remains burdened with high morbidity, with complication rates approaching 40–50% of patients. Iatrogenic complications commonly result in prolonged hospitalisation, readmission (11–25% of discharged patients), need for reoperation (9%) or interventional procedures (14%). In descending order of frequency, the commonest postoperative adverse events are delayed gastric emptying (DGE), pancreatic fistula (PF), wound infections, biliary leakage, haemorrhage, abscesses, acute pancreatitis and intra-abdominal venous thrombosis [1–3].

Multidetector computed tomography (CT) currently represents the mainstay modality to investigate the postoperative abdomen, as it can rapidly and consistently detect iatrogenic complications, thus allowing a timely and appropriate choice between conservative, percutaneous or surgical treatment. As well presented by Mauri et al. [5], interventional radiology is increasingly used and very effective to treat most PD complications, allowing imaging-guided drainage of collections and biliary leaks, transarterial control of bleeding, venous interventions and percutaneous embolisation of postoperative fistulas via trans-drainage injection of ethanol or cyanoacrilic glue [4–8].

Unfortunately, interpretation of early postoperative CT imaging is generally challenging due to the surgically altered anatomy. Aiming to improve radiologists’ familiarity with postsurgical abdominal studies, this pictorial essay reviews and illustrates the expected postoperative CT appearances and the imaging features of typical and unusual post-PD complications [9–11].

## Basics of pancreatico-duodenectomy

Most PDs are performed to manage resectable pancreatic ductal carcinoma, neuroendocrine and malignant intraductal papillary-mucinous neoplasms, cancers of the distal common bile duct (CBD), Vaterian ampulla and duodenum. Other indications include symptomatic chronic pancreatitis refractory to medical treatment and selected benign tumours not amenable to conservative surgery. The use of laparoscopy and robotic techniques is still limited in oncological pancreatic surgery [12–14].

**Fig. 1:**
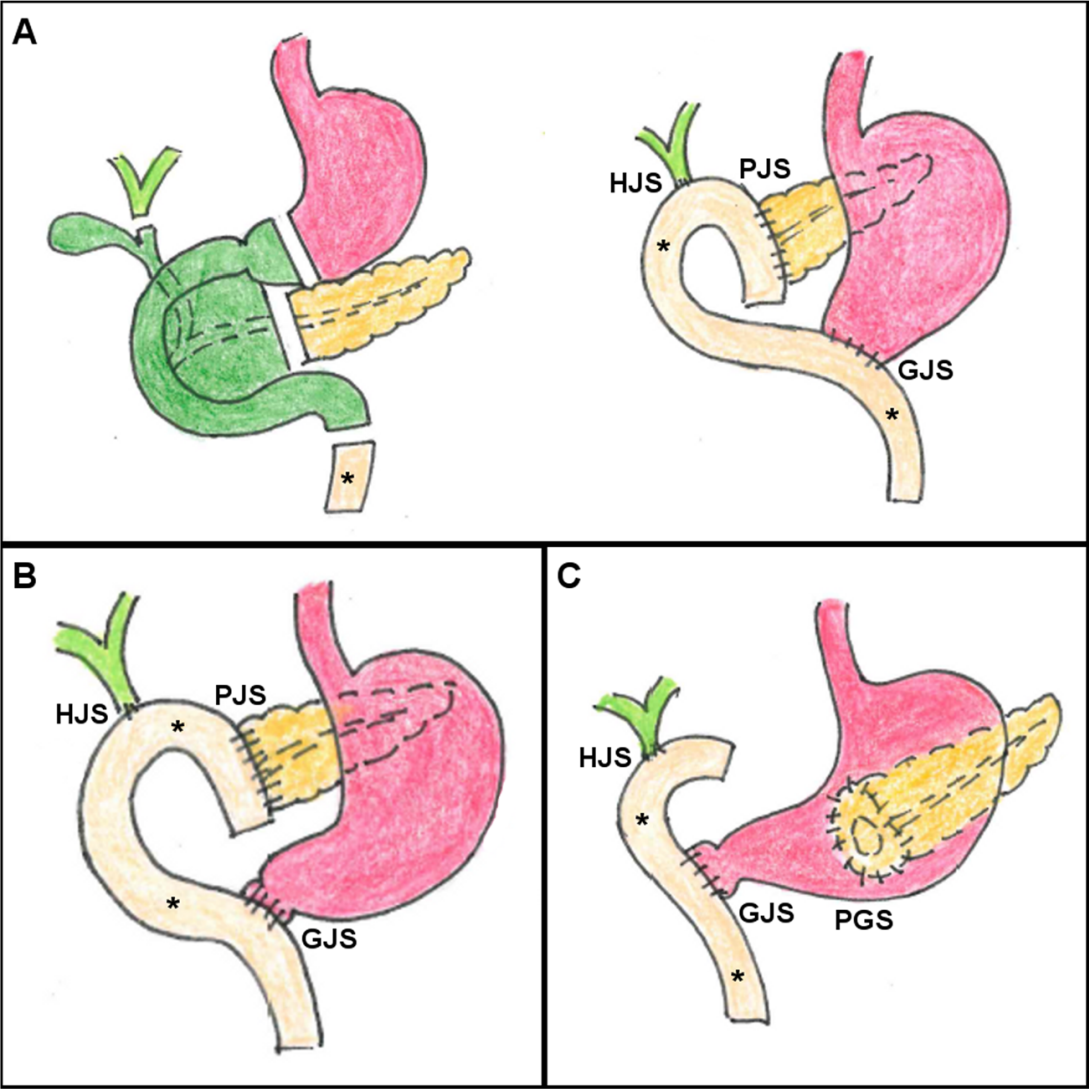
Schematic representations of postsurgical anatomy after classic Whipple (**a**), pylorus-preserving (**b**) and variant (**c**) pancreatico-duodenectomy (PD). The resected pancreatic head and neck, duodenum, choledochus and gallbladder are shown in *green* (**a**). The stomach is shown in *magenta*, the pancreatic remnant (PR) body and tail in *yellow*, the common hepatic duct and main intrahepatic branches in *pale green*. *Asterisk* indicates the mobilised jejunal loop (MJL). Note hepatico-jejunostomy (*HJS*), pancreatico-jejunostomy (*PJS*), gastro-jejunostomy (*GJS*), duodeno-jejunostomy (DJS) and pancreatico-gastrostomy (*PGS*)

The classic (Whipple’s) PD (shown in Fig. 1a) involves several steps, namely: (1) exposure of the superior mesenteric vessels and intraoperative assessment of resectability; (2) cholecystectomy; (3) transection of the distal stomach, proximal jejunum near to the ligament of Treitz and pancreatic neck; (4) regional lymph node dissection; (5) en-bloc removal of the pancreatic head, neck and uncinate process along with the duodenum and choledochus. Compared to the above-described operation, the Traverso-Longmire pylorus-preserving technique (Fig. 1b) spares the gastric antrum [12–14].

Surgical reconstruction requires creation of: (1) an end-to-side anastomosis between the mobilised jejunal loop (MJL) and pancreatic duct [pancreatico-jejunostomy (PJS)]; (2) an end-to-side anastomosis between common hepatic duct and MJL [hepatico-jejunostomy (HJS)]; (3) either gastro-jejunostomy (GJS) in Whipple PD or duodeno-jejunostomy (DJS) in pylorus-preserving PD. Alternatively, some centres perform a variant technique (Fig. 1c), in which the PR and pancreatic duct are connected to the dorsal aspect of the stomach [pancreatico-gastrostomy (PGS)]. If required by venous invasion, experienced surgeons can also perform reconstructions or grafting of the superior mesenteric and portal veins [12–14].

There are no relevant differences in complication patterns and rates between the three PD variants [14, 15]. General risk factors for increased morbidity include prolonged duration of surgery, significant intraoperative blood loss and high body-mass index (particularly regarding high-grade PF). The effect of advanced age is controversial: although overall complication rates are not substantially increased, mortality and risk of pneumonia are higher in elderly patients [16–20].

## Early post-pancreatico-duodenectomy CT

### Indications

Within the first 2 or 3 postoperative days after PD, the commonest indications for CT imaging include suspected early haemorrhage, peritonitis, physical and laboratory signs of systemic inflammation. Post-PD bleeding may be either intraluminal or extraluminal: the latter heralded by blood from drainage, nasogastric tube or abdominal incision site. On the other hand, the less common intraluminal haemorrhage manifests with haematemesis or melaena. In both situations, variable degrees of abdominal pain, signs of haemodynamic impairment and dropping haematocrit are present. Unfortunately, clinical and laboratory findings may not accurately reflect the true entity of bleeding [6, 21].

After the early postsurgical hospitalisation, the usual indications for CT imaging include suspected DGE with persistent need for nasogastric intubation, peripancreatic drainage yielding high-amylase fluid consistent with PF, increasing leucocyte count and C-reactive protein levels, as well as physical and laboratory signs of delayed haemorrhage. In our experience, surgeons increasingly think that physical findings, abdominal pain and distension are relatively insensitive and rely on routine postoperative CT imaging [6, 21].

### Acquisition technique

Due to high prevalence of pleuropulmonary changes, we suggest to routinely include the lung bases in postsurgical abdomen/pelvis CT studies. Borrowing from experience after gastric surgery, oral administration of diluted water-soluble contrast medium (CM) a few minutes prior to CT has been suggested to improve identification of bowel loops and diagnostic confidence in the diagnosis or exclusion of anastomotic leaks. However, in the setting of PD surgery, most centres—including ours—discourage the use of oral CM, since it may cause beam-hardening artefacts and hamper detection of haemorrhage. Furthermore, recently operated patients are often unwell and not willing or able to swallow, particularly those with a distended stomach secondary to DGE [9–11].

**Fig. 2:**
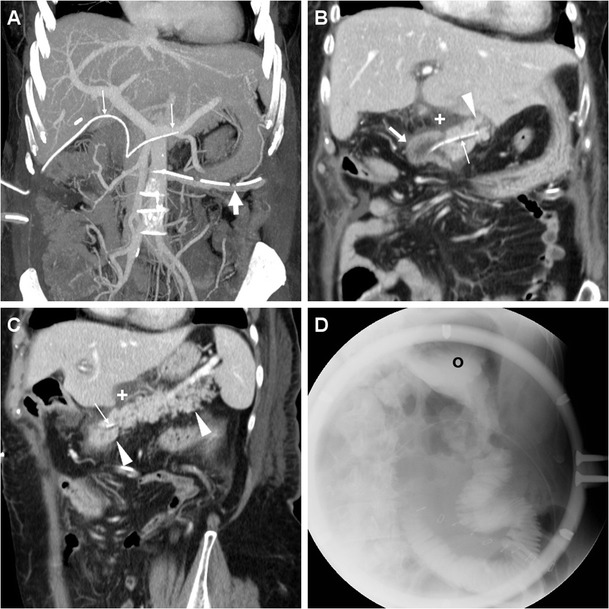
Expected CT findings following uncomplicated PD performed for malignant intraductal mucinous-papillary tumour of the pancreatic head in a 69-year-old woman. Coronal maximum intensity projection (MIP) reconstruction (**a**) showed presence of a left-sided abdominal drainage tube (*thick arrow*) and of an externally draining trans-anastomotic stent (*thin arrows*). Focused coronal (**b**) and oblique-coronal (**c**) contrast-enhanced images showed minimal fluid (+) abutting the PJS, normal appearance of the PR (*arrowheads*) and MJL (*arrows*). In the same patient, fluoroscopy (**d**) showed normal flow of oral contrast medium (CM) from the gastric remnant (o) through the GJS

Obtaining precontrast scans is useful to identify external drainage tubes, metallic staples, trans-anastomotic stents and hyperattenuating fresh blood in the abdomen or gastrointestinal lumen. Study review at lung or bone window settings improves visualisation and quantification of residual intraperitoneal air. Unless contraindicated by allergy or renal failure, enhancement by intravenous iodinated contrast medium (CM) is warranted after recent PD. We recommend to acquire post-PD studies using a typical pancreatic CT protocol, including a late-arterial phase (acquired either 35–40 s after start of intravenous contrast injection or 10–15 s after bolus tracking using a region of interest in the abdominal aorta and 110-HU threshold) and a portal-venous phase (using a fixed 80-s delay). Additionally, when clinical or laboratory findings suggest possible bleeding, adding an early arterial-phase acquisition is beneficial to detect active haemorrhage and to provide a vascular roadmap to the interventional radiologist by reconstructing maximum intensity projection (MIP) CT-angiography images. Reconstructing thick-slab maximum-intensity (MIP) images (Fig. 2a) may be helpful to visualise the presence, number, course and distal tip position of abdominal and peripancreatic surgical drains, and to improve detection of CM extravasation indicating active bleeding [10, 11, 22].

### Normal postsurgical findings after pancreatico-duodenectomy

A checklist for interpretation of early post-PD CT studies is provided in Table 1. Such as after most major abdominal surgeries, pleural effusion, atelectasis and pneumonia are commonly encountered, particularly in elderly men with chronic obstructive lung disease.

**Table 1:** Checklist for interpretation of early CT after pancreatico-duodenectomy (PD)

Feature	Comments
Report pleuropulmonary changes (such as atelectasis, pneumonia, pleural effusion) at lung bases	Particularly common in elderly patients
Externally draining tubes present?	Use thick-slab maximum-intensity projection (MIP) reconstructions Report presence, number, course and distal tip position
Identify - pancreatic remnant (body and tail) - main pancreatic duct (MPD) - either pancreatico-jejunostomy (PJS) or pancreatico-gastrostomy (PGS)	Best visualised in oblique-coronal images Assess calibre Assess integrity, presence of internal or external trans-anastomotic stents
- mobilised jejunal limb	Identified by valvulae conniventes and tubular configuration on coronal images; mural oedema is generally normal
Identify - hepatico-jejunostomy (HJS) - either gastro-jejunostomy (GJS) or duodeno-jejunostomy (DJS) - gastric dilatation	Pneumobilia and/or mild biliary tract dilatation are usually normal Respectively after Whipple and pylorus preserving PD Suggest delayed gastric emptying (optional fluoroscopy for confirmation)
Identify fluid collections and air - surgical bed, abutting the PJS - subhepatic/right-sided - surrounding PR - pneumoperitoneum/peritonitis	Report as consistent with a clinical/laboratory diagnosis of pancreatic fistula (fat stranding, mild non-demarcated fluid, small lymphadenopathies are usually normal) Suggest bile leakage Suggest acute pancreatitis Mild residual air within 3 days is usually normal Persistent or abundant pneumoperitoneum, diffuse ascites, enhancing peritoneal serosa suggest peritonitis from major anastomotic leakage
Search for bleeding - intraluminal in jejunum - extraluminal - hemoperitoneum	Use MIP reconstructions Compare precontrast, arterial- and portal venous phase images Always scrutinise the gastroduodenal artery “stump”
Assess patency of splenic, portal and mesenteric veins	For postoperative thrombosis, favoured by venous resections or graft insertion
Scrutinise laparotomic incision site	For fluid or abscess collections consistent with wound infection

**Fig. 3:**
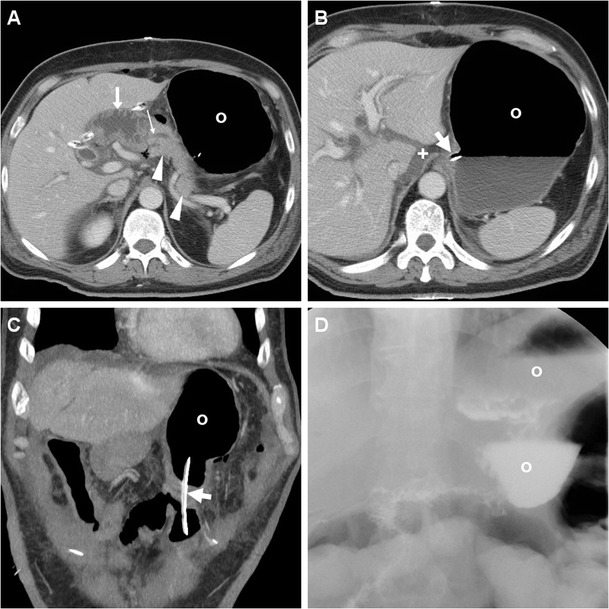
Expected CT findings following uncomplicated PD performed for pT3N0 adenocarcinoma of the Vaterian ampulla. Contrast-enhanced CT (**a**-**c**) showed distended stomach (o) with stagnant fluid despite nasogastric intubation (*thick arrows*), consistent with delayed gastric emptying (DGE); normal appearance of the PR (*arrowheads*), the MJL and the PJS with low-attenuation stent (*thin arrow*) in the main pancreatic duct (MPD). In the coronal image (**c**) the nasogastric tube (*thick arrow*) courses through the GJS. Fluoroscopy (**d**) confirmed DGE with persistently dilated stomach (o) with stagnant oral CM

**Fig. 4:**
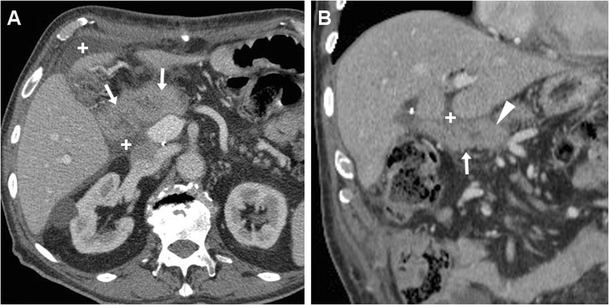
**a, b** Transient oedema of the MJL after uncomplicated PD in a 72-year-old man. Contrast-enhanced CT showed mildly thickened MJL walls (*arrows*). Note PR (*arrowhead*), minimal subhepatic fluid (+)

**Fig. 5:**
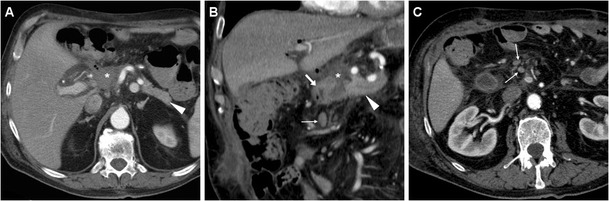
**a-c** Postoperative pancreatic fistula (PF) after Whipple PD for pT3N1 pancreatic ductal adenocarcinoma in a 72-year-old man with persistent output and increasing amylase levels in fluid from drainage. On postoperative day 9, CT (Fig.3) showed a mixed-attenuation collection (*) extending upwards from the PJS, between the PR (*arrowheads*) and MJL (*arrows*), consistent with clinico-biological diagnosis of PF. These CT changes ultimately resolved on conservative treatment. Note intrahepatic pneumobilia (in **b**), some small-sized lymph nodes (*thin arrows*) surrounding the superior mesenteric vessels. (Partially adapted with permission from Tonolini [25])

The pancreatic remnant (PR) corresponding to the body and tail is best assessed using an oblique-coronal plane (Fig. 2). Although not supported by scientific evidence [23, 24], externally draining (Fig. 2) or internal trans-anastomotic stents (Fig. 3) may be placed intraoperatively: their presence further eases identification of the PJS and of the residual main pancreatic duct (MPD). The MJL is anastomosed to the right side of the PR, generally oriented horizontally and best recognised in coronal images (Figs. 2 and 3). Characterised by the presence of valvulae conniventes, the MJL should not be misinterpreted as blood or abscess collection. In normal conditions, the MJL may show thickened oedematous walls and bright mucosal enhancement (Fig. 4). The HJS or biliary-enteric anastomosis is often challenging to identify and best recognised in a coronal orientation (Fig. 3). Although less common than in the past, pneumobilia (Fig. 5b) should not be considered abnormal. Mild dilatation of the common hepatic duct requires correlation with laboratory tests [9, 11, 22, 26].

**Fig. 6:**
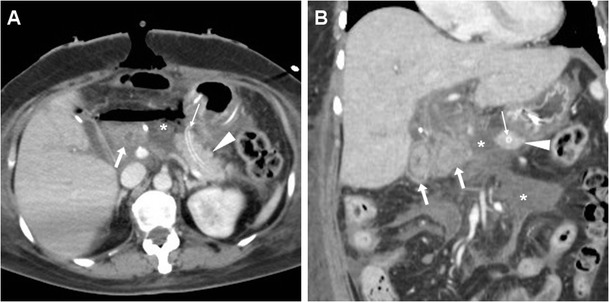
**a, b** Dehiscence of the PJS with peritonitis in a 59-year-old woman with shock, sepsis and peritonitis 48 h after PD performed for a benign tumour of the Vaterian ampulla. CT showed discontinuity between the PR (*arrowheads*) with MPD stent (*thin arrows*) and MJL (*arrows*), filled by a large air-fluid collection (*) extending in the mesentery. Emergency relaparotomy was performed, with creation of a gastro-pancreatic anastomosis

Observed in almost 50% of early post-PD studies, usual CT findings which should not reported as abnormal include oedematous fat stranding in the surgical bed, scanty fluid (Fig. 2) extending to the lesser sac, mesentery and subhepatic space, soft-tissue “cuffing” surrounding the superior mesenteric vessels, tiny sub-centimetre lymph nodes in the central mesentery (Fig. 5c). Within the first 3 postoperative days, some residual intra-abdominal air is commonly observed, either in a free non-dependent distribution or as bubbles radiating from the site of operation. However, in our experience, persistent or abundant pneumoperitoneum, diffuse ascites and enhancing peritoneal serosa should be viewed with caution as they may correspond to peritonitis from major anastomotic leakage (Fig. 6) requiring reoperation [10, 11, 22].

### Delayed gastric emptying

A dilated stomach with stagnant fluid and/or oral CM (Fig. 3) is the hallmark of DGE, which remains an unsolved problem after both classic and pylorus-preserving PD. Although a consensus definition is lacking, DGE with persistent need for nasogastric intubation occurs in 20-50% of patients, most often in the elderly, and may worsen the nutritional state and prolong hospitalisation. The exact mechanism is unknown, but likely involves loss or damage of autonomic innervation of the stomach, and may be decreased by special surgical techniques with subtotal stomach preservation and antecolic reconstruction [17, 27, 28].

Located in variable positions according to surgeons’ preference, the GJS (Fig. 3c) is best viewed in the coronal orientation and sometimes indicated by metallic stapling along the gastric suture. Traditionally, contrast fluoroscopic studies were use to assess position, patency and integrity of the GJS (Fig. 2d) and to detect delayed or absent emptying of the residual stomach consistent with DGE (Fig. 3d) [10, 11, 22].

## Postoperative pancreatic fistula

Defined by leaking pancreatic secretions at the PJS, PF represents the single most important cause of post-PD morbidity with an overall incidence of 17–30%. PF is more frequent in obese individuals and following PD for ampullary and duodenal cancers rather than for pancreatic tumours [19, 28–31]. Patients with “soft” pancreatic texture reflecting fatty infiltration are more prone to develop PF. At CT, an increased risk of PF may be predicted by high visceral fat area, low attenuation of abdominal viscera and paraspinal muscles, large pancreatic volume and small (<3 mm) pancreatic duct calibre [32–36].

According to the International Study Group on PF, this condition is diagnosed on the basis of “any measurable output from peripancreatic drainage on or after postoperative day 3 with amylase content >3 times the serum amylase”, alternatively at reoperation or percutaneous drainage. In the recent 2016 re-definition, grade A is now termed “biochemical leak” and no longer considered a true PF. The clinically significant grades B and C PF are respectively defined as “requiring modification in postoperative management (drainage left in place >3 weeks or repositioned through endoscopic or percutaneous procedures)” and “requiring reoperation or causing single or multiple organ failure”. Whereas the overall PF-related mortality is approximately 1%, grade C is associated with 25.7% mortality [19]. Importantly, even low-grade PF is strongly associated with a higher incidence of reoperation and of other non-fistulous complications (incidence 51% versus 21% in patients without PF) such as pancreatitis, abscess formation, haemorrhage, bile leakage, wound and systemic infection [19, 29–31].

The above-mentioned clinico-biological criterion diagnoses PF on average 7 days after PD with 70–75% sensitivity, but is not sufficiently reliable in the early postoperative period. Unfortunately, PF may be clinically silent or manifest after discharge or resumption of oral feeding: therefore, the use of CT is valuable to decrease the occurrence of occult or delayed PF [37].

**Fig. 7:**
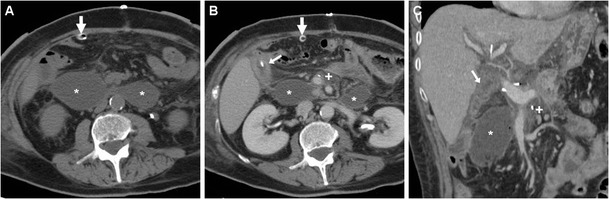
Non-infected postoperative collections in a 71-year-old man following PD for duodenal adenocarcinoma, complicated by intraoperative haemorrhagic shock. During prolonged hospitalisation, unenhanced (**a**) and postcontrast (**b**, **c**) CT images showed “saddlebag”-shaped retroperitoneal collection (*) with homogeneous fluid attenuation, thin walls. Note usual appearance of the MJL (*arrows*), scanty mesenterial fluid (+), drainage still in place (*thick arrows*). The patient ultimately recovered without additional procedures

**Fig. 8:**
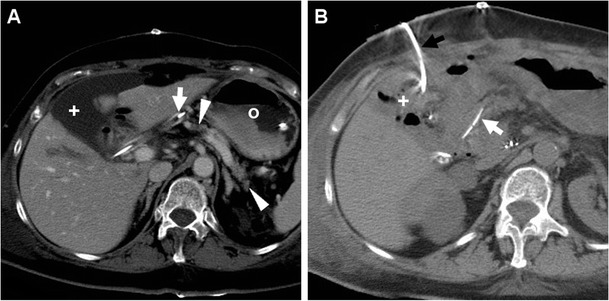
Bile collection found 4 days after PD for distal common bile duct (CBD) cholangiocarcinoma in an 83-year-old woman with biliary leakage from the laparotomic incision site. Contrast-enhanced CT (**a**) showed distended stomach (o) consistent with DGE, external drainage (*thick arrows*) in place, normal PR (*arrowheads*), and a non-encapsulated 10 × 5 cm water-attenuation collection (*) in the gallbladder fossa, which was treated with percutaneous drainage. Unenhanced follow-up CT (**b**) showed minimal residual fluid and air (+) in the site of the biloma

**Fig. 9:**
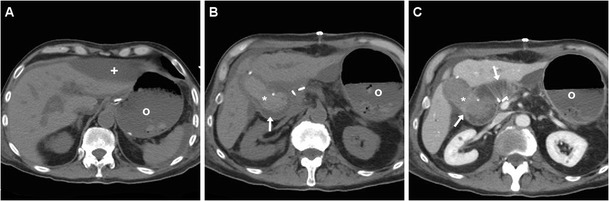
Biloma and intraluminal haemorrhage in a 72-year-old man after pylorus-preserving PD for CBD cholangiocarcinoma and initial diagnosis of postoperative PF treated conservatively. On postoperative day 16, precontrast (**a**) and contrast-enhanced (**b**, **c**) CT showed fluid-attenuation collection (+) located ventrally and inferiorly to the left liver lobe, consistent with bile; distended stomach (o) with fluid indicating DGE, hyperattenuating blood (*) in the distended MJL (*arrows*). Without CT evidence of active bleeding, the patient was treated conservatively with blood transfusions

The presence of a focal collection at the surgical site, particularly abutting the PJS, should be reported as highly suggestive or consistent with a diagnosis of PF (Fig. 5). The variably-shaped PF-related collections generally show fluid-like or slightly increased attenuation, and may occasionally contain gas bubbles or show peripheral enhancement. Routine CT screening on day 7 for occult PF in patients at high risk resulted in diagnosis of PF in 54% of patients, with 63% sensitivity and 83% specificity. In that study, false-positive collections were usually smaller than 2 cm, never contained air bubbles and disappeared at follow-up scanning. Conversely, false negative CTs were secondary to drainage tube positioned immediately adjacent to the PJS. PF should be differentiated from PJS dehiscence (Fig. 6) and from other collections which do not fulfil the biochemical criterion (Fig. 7), including bilomas (Figs. 8, 9). Worrisome features for dehiscence include wide-open PJS, increasing volume of collections, abundant gas and development of peritonitis (Fig. 6) [9–11, 37].

The majority (90%) of PF occurrences can be managed non-surgically with parenteral nutrition, octreotide and antibiotics until fistula closure, plus percutaneous drainage of major biliary collections and abscesses. Small collections that are not amenable to aspiration should be considered as probable PF and monitored until resolution [5, 8, 23, 29].

## Miscellaneous complications

The other important post-PD complications include postoperative haemorrhage (4–16% incidence), wound infection, intra-abdominal and hepatic abscesses (3–8%), biliary leakage (1–5%), acute pancreatitis of the PR (2–3%), thrombosis of the portal or superior mesenteric veins (particularly after complex venous reconstructions) and visceral ischaemia (below 1%) [28, 30].

### Biliary leaks

Leakage of bile primarily results from technical failure of the HJS. Although CT cannot assess for sure whether fluid leaks from the PJS or HJS, biloma is suggested by a homogeneous, non-enhancing water-attenuation collection, which generally lies in the subhepatic space or right hemiabdomen (Figs. 8 and 9) [10, 11, 22].

In the vast majority of cases, bile collections are successfully managed without surgery, often with percutaneous drainage (Fig. 8) until spontaneous closure of leakage occurs [5, 8].

### Abscesses

**Fig. 10:**
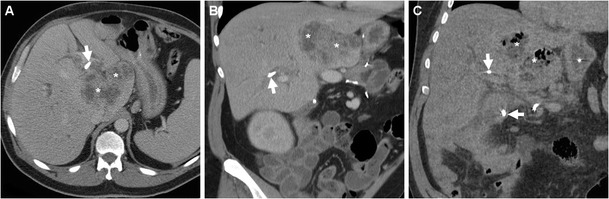
Liver abscess in a 55-year-old man following PD performed at another hospital and complicated by sepsis. CT (**a**, **b**) showed development of a multilocular mass with peripheral and septal enhancement (*) in the caudate lobe. Before the patient passed away, repeated unenhanced CT 72 h later (**c**) showed development of gas in the liver abscess (*). Note external biliary drainage (*thick arrows*)

Infected collections may develop secondary to either superinfection of an acute postoperative fluid collection (including these from PF) or leaking GJS/DJS. The well-known hallmark of an abscess is a complex collection with central hypoattenuation and thick peripheral and septal enhancement. Sepsis may even progress to involve the liver (Fig. 10), either by contiguity or by ascending biliary infection [10, 11, 22].

### Bleeding

Post-PD haemorrhage accounts for almost one-third of the in-hospital mortality. Early bleeding develops within 24 h from surgery, is generally severe and most usually results from inadequate ligation of the gastroduodenal artery (GDA). Less common sites of bleeding include the common hepatic, right gastric and peripancreatic arteries. Conversely, the more frequent late bleeding occurs after a variable delay (median 33 days, up to 10 weeks) and is preceded by PF, anastomotic leak or intra-abdominal sepsis in approximately one-half of cases [6, 21, 28].

**Fig. 11:**
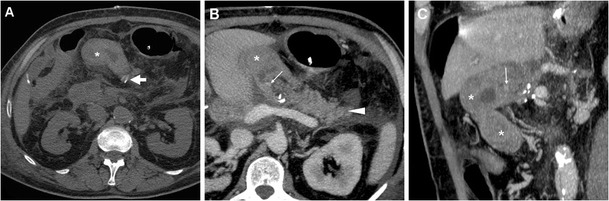
Extraluminal haemorrhage in a 70-year-old man after PD for pT3N1 carcinoma of the Vaterian ampulla, who experienced acute abdominal pain on postoperative day 3: precontrast (**a**) showed hyperattenuating blood (*) extending ventrally from the surgical bed. Contrast-enhanced CT with thin-slab MIP reconstructions (**b**, **c**) showed a tiny focus of CM extravasation (*thin arrows*) consistent with active bleeding. Note normal-appearing PR, drainage tube (*thick arrows*). Relaparotomy was required to stop bleeding from a small artery at the site of pancreatic resection

**Fig. 12:**
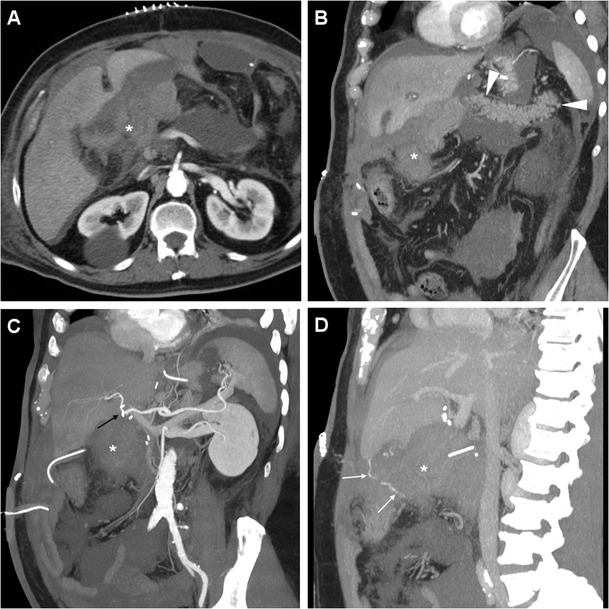
Venous extraluminal haemorrhage in a 74-year-old man following pylorus-preserving PD for pT3N1 CBD adenocarcinoma, suffering from hypotension, abdominal pain and blood from drainage tube on postoperative day 7. Urgent CT showed haemoperitoneum, fresh blood (*) extending from the surgical bed in the subhepatic space and mesentery, normal appearance of RP (*arrowheads* in **b**). CT-angiography MIP reconstructions (**c**) did not detect active arterial bleeding or pseudoaneurysms, particularly at the gastroduodenal artery “stump” (*thin black arrow*). In the venous phase (**d**) serpiginous CM extravasation (*thin arrows*) was detected. Emergency surgery was required to control oozing venous bleeding at the transverse mesocolon. (Partially adapted from Tonolini [38])

**Fig. 13:**
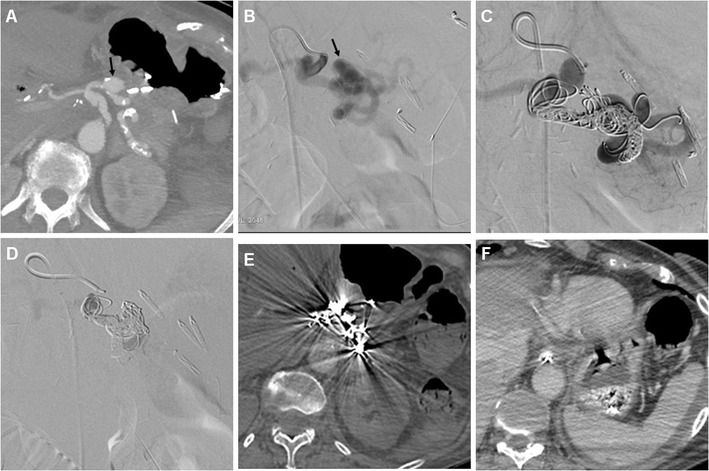
Endovascular treatment of postsurgical pseudoaneurysm in a 77-year-old woman. Three days after PD for pancreatic head adenocarcinoma, emergency CT was performed to investigate abdominal pain, blood from surgical drainage and dropping haemoglobin (>3 g/dL within 12 h). The pseudoaneurysm (*black arrow*) of the splenic artery depicted by MIP CT reconstruction (**a**) was confirmed angiographically (**b**). Embolisation was performed with a “sandwich technique”, placing coils both distally and proximally to the pseudoaneurysm, plus 0.2 ml of glue, due to persistent flow through the coils (**c**). Final angiogram (**d**) confirmed complete exclusion of the pseudoaneurysm. Follow-up CT confirmed successfully treated pseudoaneurysm (**e**) and normal perfusion of the spleen (**f**) supplied by collaterals

Bleeding may develop either intraluminally or extraluminally: in haemodynamically stable patients who do not require immediate laparotomy, CT reliably detects the presence of high-attenuation fresh blood in the jejunal lumen (Fig. 9), surgical bed or mesentery (Figs. 11, 12) and peritoneal cavity (Fig. 12). CT angiography with MIP reconstructions effectively shows the postoperative vascular anatomy, and may precisely identify the presence of CM extravasation in either the arterial (Fig. 11) or venous (Fig. 12) phase, indicating active bleeding. Being the commonest site of early bleeding, the GDA “stump” at origin from the hepatic artery (Fig. 12c) should be carefully scrutinised. Sometimes, perfused vascular outpouchings representing pseudoaneurysms (Fig. 13a) may be recognised at the site of arterial injury [6, 9–11, 39].

Rapid CT imaging diagnosis is crucial to dictate and guide transarterial embolisation, which is increasingly preferred as first-line treatment with 75–85% success rates [5–7, 21]. Selective embolisation of pseudoaneurysms (Fig. 13b-f) is associated with a higher recurrence of bleeding compared to endovascular trapping of the hepatic artery [40].

### Postoperative pancreatitis

**Fig. 14:**
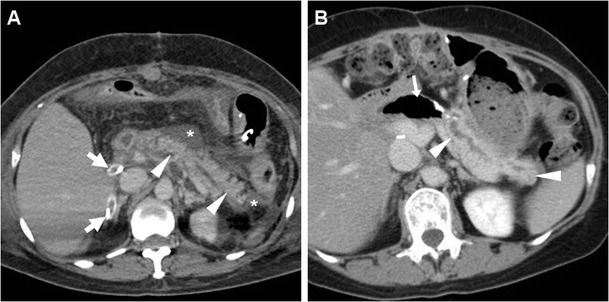
Delayed postoperative acute pancreatitis in the same 59-year-old woman as in Fig. 6, after early PJS dehiscence and surgical reintervention. Nearly a month after PD, CT (**a**) showed fluid (*) collecting around the medium-sized and normally enhancing RP, which ultimately resolved at follow-up (**b**) with residual MPD dilatation. Note drainage tubes (*thick arrows*), air-containing MJL (*arrow* in **b**)

Differentiating acute pancreatitis of the PR from usual inflammatory changes and fluid in the surgical bed may be challenging. Furthermore, elevated serum markers may also result from surgical manipulation. The key appearance consistent with a diagnosis of pancreatitis is disproportionate distribution of inflammatory changes and fluid surroundings the PR rather than in the surgical bed (Fig. 14) [10, 11, 22].

### Ischaemic complications

Occasionally, ischaemia of the liver, stomach and/or spleen may develop after PD secondary to either inadvertent injury, ligation or clamping of the hepatic artery or celiac trunk during surgical dissection, or impaired visceral perfusion in patients with pre-existing conditions such as atherosclerosis, median arcuate ligament compression or fibromuscular dysplasia. Preoperative recognition and appropriate management of underlying haemodynamically significant arterial strictures is beneficial to prevent these lethal (50–83% mortality) complications [28, 41–43]. The resulting CT appearances include devascularisation of the gastric wall or left liver lobe [9–11].

## Conclusions

Following PD, multidetector CT rapidly provides a comprehensive visualisation of the operated abdominal compartment, and represents a consistent basis for triage of iatrogenic complications and correct choice between conservative, interventional or surgical treatment. Understanding the surgically altered anatomy and awareness of expected postoperative appearances is crucial to correctly recognise and classify complications.
